# Should the poor have no medicines to cure? A study on the association between social class and social security among the rural migrant workers in urban China

**DOI:** 10.1186/s12939-017-0692-x

**Published:** 2017-11-07

**Authors:** Ming Guan

**Affiliations:** 1 Family Issues Center, Xuchang University, Road Bayi 88, Xuchang, Henan China; 2 School of Business, Xuchang University, Road Bayi 88, Xuchang, Henan China

**Keywords:** Social class, Rural migrant workers, Social security inequity, Medical inequity, Reimbursement rejection

## Abstract

**Background:**

The rampant urbanization and medical marketization in China have resulted in increased vulnerabilities to health and socioeconomic disparities among the rural migrant workers in urban China. In the Chinese context, the socioeconomic characteristics of rural migrant workers have attracted considerable research attention in the recent past years. However, to date, no previous studies have explored the association between the socioeconomic factors and social security among the rural migrant workers in urban China. This study aims to explore the association between socioeconomic inequity and social security inequity and the subsequent associations with medical inequity and reimbursement rejection.

**Methods:**

Data from a regionally representative sample of 2009 Survey of Migrant Workers in Pearl River Delta in China were used for analyses. Multiple logistic regressions were used to analyze the impacts of socioeconomic factors on the eight dimensions of social security (sick pay, paid leave, maternity pay, medical insurance, pension insurance, occupational injury insurance, unemployment insurance, and maternity insurance) and the impacts of social security on medical reimbursement rejection. The zero-inflated negative binomial regression model (ZINB regression) was adopted to explore the relationship between socioeconomic factors and hospital visits among the rural migrant workers with social security.

**Results:**

The study population consisted of 848 rural migrant workers with high income who were young and middle-aged, low-educated, and covered by social security. Reimbursement rejection and abusive supervision for the rural migrant workers were observed. Logistic regression analysis showed that there were significant associations between socioeconomic factors and social security. ZINB regression showed that there were significant associations between socioeconomic factors and hospital visits among the rural migrant workers. Also, several dimensions of social security had significant associations with reimbursement rejections.

**Conclusions:**

This study showed that social security inequity, medical inequity, and reimbursement inequity happened to the rural migrant workers simultaneously. Future policy should strengthen health justice and enterprises’ medical responsibilities to the employed rural migrant workers.

**Electronic supplementary material:**

The online version of this article (10.1186/s12939-017-0692-x) contains supplementary material, which is available to authorized users.

## Background

As a research topic, social class and medical care had been linked since 1960s [1, 2]. Social class worked as a predictor of choice of health care provider [3]. Also, social class might explain the differences in clinical outcomes [4]. Clinically, social class had association with the use of dental care under prepayment [5], life expectancy and overall mortality [6]. Remedially, there was a significant correlation between assignment of patients to therapy and social class in the case of unemployed status [7]. Regarding health care, due to a relative lack of psychological, social and financial resources, people with low socioeconomic status coped less effectively with sudden changes in the provision of health services [8]. Within the National Health System in Spain, social inequalities were still evident for some curative and preventive services [9]. Additionally, there existed different levels of utilization of health services by social class [10]. However, the existing literature on the relationship has largely been limited to migrants within the western countries, which made it difficult to generalize the results in Asian situations and to reflect the relationships between key factors in urban China comprehensively.

With low social class in urban China, rural migrant workers came from the major impoverished villages in remote regions featured harsh natural conditions. The poor economic conditions, local people’s ignorance, backwardness, conservatism, and lack of desire to change the status quo were major obstacles to local social and economic development [11]. Thus, they were marginalized in national hierarchical structures in rural China.

In the cities, most of rural migrant workers experienced discrimination in daily life and perceived social inequity which had a significant influence on their mental health [12]. Social stigma against rural migrants was common in urban China, which leaded to negative health consequences [13]. Additionally, discriminatory experience had significant negative effects on quality of life among the rural migrant workers [14, 15]. In particular, quality of life of female rural migrant workers was lower than Chinese female norms in the Shenzhen city [16]. Thus, they were socially marginalized in urban hierarchical structures in modern China.

A substantial global literature suggested that migration across health and disease disparities influences the epidemiology of certain diseases globally and in nations receiving migrants [17].Previous research also documented that the experience of discrimination and perceived social inequity encountered by rural migrants in urban China may cause mental illnesses [18]. But to date, no studies explored medical inequity caused by social inequity among the rural-urban migrants in China. This study tried to fill in the gaps.

Rural migrant workers accounted for a disproportionate burden of occupational injury morbidity and mortality in China [19]. But, only workers in the state-sector were covered by social security before China’ entry into the World Trade Organization [20, 21]. Thus, rural migrant workers were dropped out of the social security program. Moreover, the health problems resulted from internal migration posed particular demands on health care systems in China [22]. China’s disparity in health insurance coverage [23] and inequity of health care financing distribution [24] were partly influenced by rural-urban migration. Furthermore, quality of primary health care delivered to migrants was less satisfactory than to local residents in terms of attitude to health workers and waiting time [25]. A moral claim had been commonly accepted that the current insurance system must include migrants in order to achieve universal coverage [26]. Till now to my best knowledge, no studies have examined the relationship between socioeconomic factors and social security among the rural migrants in urban China. This study intends to fill this gap.

The early studies reported Chinese relationship between social security and medical reimbursement. For example, the coverage of medical reimbursement was small in the cases of seasonal influenza vaccination [27] and new rural cooperative medical scheme policy in rural China [28]. But, the relationship was not discovered among the rural migrant workers in urban China. As a result of extremely uneven distribution of household assets in China, The growing inequalities in healthcare and the increasing financial burdens presented by medical expenditure have been a source of social discontentment. Thus, it was important to reveal the link between social class and social security.

In this study, the topics above would be analysed with a comprehensive data from a community-based survey conducted in Pearl River Delta, Guangdong Province of China. It is one of the largest destination regions that Pearl River Delta received approximately 51.99 million rural migrant workers in China. Numerous studies sampled in the region had described the marginalized living and work conditions confronting rural migrant workers, such as experiencing circular migration [29], prevalence of depressive disorder [30, 31], lack of work injury insurance provision [32], poor living conditions and inattention to health [33], hard environment [34], peasant identity [35], lack of occupational health services [36], low rates of supply and use of personal protective equipment [37], unequal socioeconomic distribution of health [38], impairment in health-related quality of life and less social support [39], and discrimination by urban locals [40]. As cheap labors, health status of the rural migrant workers often was neglected by urban bosses under abusive supervision. The workers with diseases were often unemployed by their employers in order to reduce employment cost. Since 2003, it has attracted the public attention that the rural migrant workers were often unpaid. Thus, it was a reasonable guess to say that rejection of medical reimbursement could happen to the venerable persons. Till now, no published studies examined the relationship between social security and medical reimbursements in the region. This study will bridge this gap.

Therefore, the aim of the present study was to assess the association between social security and social class among the rural migrant workers. The logistic regressions and the zero-inflated negative binomial regression (ZINB regression) models were adopted here to discover how difficult the rural migrant workers with lower social class were risky of social security inequity, medical inequity and reimbursement inequity.

## Methods

### Data source

Data were used from a regionally representative sample 2009 Survey of Migrant Workers in Pearl River Delta (SMWPRD, http://css.sysu.edu.cn/). SMWPRD included questions about social security, social class, and further about background demographic characteristics including detailed questions about income and occupation. This survey was organized by Professor Linping Liu in Center for Social Survey at Sun Yat-sen University, which was funded by Department of philosophy and Social Sciences, Ministry of Education in China (project number: 2009JYJR007). A total of 1766 respondents (954 males and 812 females) completed paper copies of the questionnaire. The non-response rate was 2.2%. On the basis of proportion of rural-urban migrants in the population in nine cities in the Pearl River Delta, this survey allocated questionnaires controlling the distribution of gender, industries and regions. The distribution of sampled cities was Guangzhou (16.87%), Shenzhen (23.61%), Zhuhai (4.93%), Foshan (8.72%), Zhaoqing (3.45%), Dongguan (29.84%), Huizhou (3.51%), Zhongshan (5.61%), and Jiangmen (3.45%).

One-thounsand seventy six respondents responded to the question of hospital visits. Then, it can speculate that the zeros in the hospital usage variable may represent the youth who were healthier and may not have needed health services in the last year. The excluded respondents are those who did not reply to the medical expenditure. Thus, the 848 needed respondents came from Guangzhou (16.27%), Shenzhen (22.17%), Zhuhai (4.95%), Foshan (9.20%), Zhaoqing (3.66%), Dongguan (27.00%), Huizhou (4.48%), Zhongshan (8.14%), and Jiangmen (4.13%). Similarly, it can speculate that part of migrant workers who need medical care went to pharmacy shop for medicine rather than hospital for doctors. Thus, number of hospital visits can show medical inequality among the 846 respondents.

### Sample locations

**Fig. 1:**
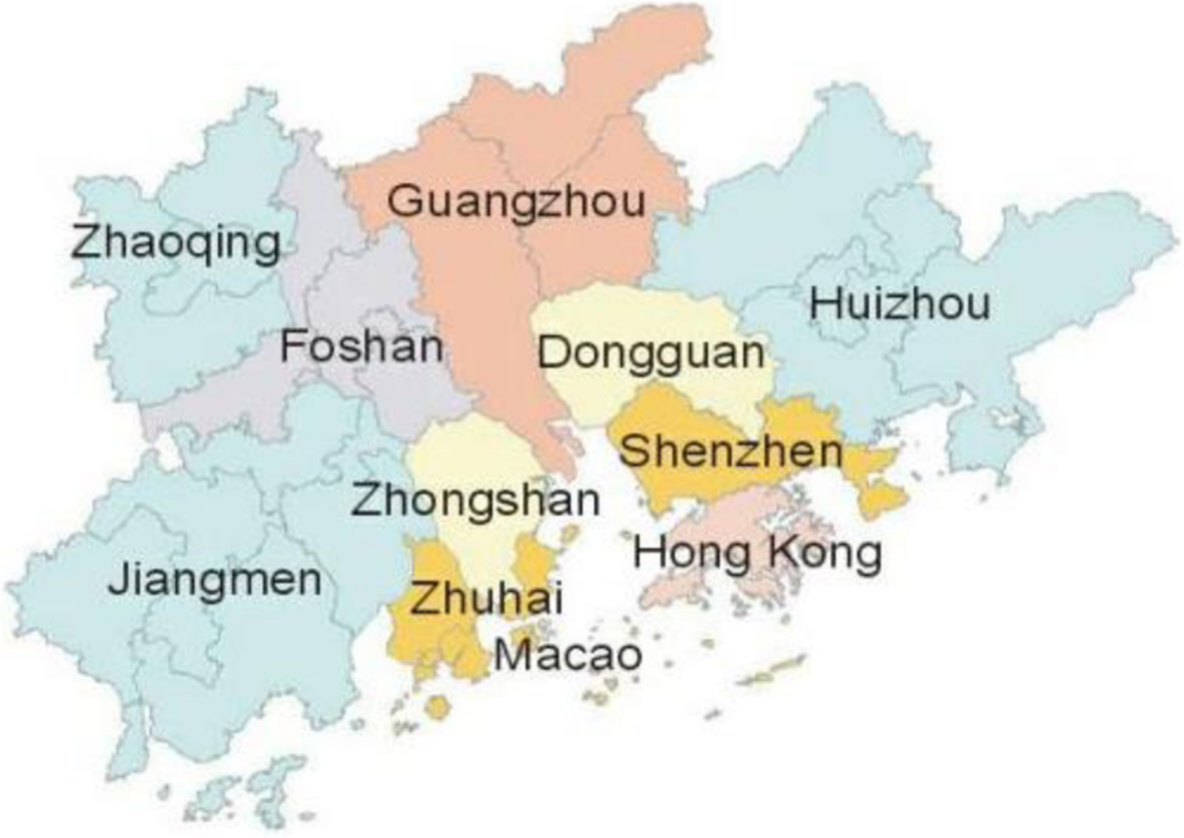
Pearl River Delta

See Fig. 1. As one of the fastest growing regions in China, Pearl River Delta has attracted a large and mobile migrant working population mainly coming from the inland areas. In the region, the abundance of employment opportunities has been created by numerous labour-intensive small- and medium-size enterprises.

### Main variables

Dependent variables came from social security. The question on social security was “Does your firm supply the following social security?” Response options were sick pay, paid leave, maternity pay, medical insurance, pension insurance, occupational injury insurance, unemployment insurance, and maternity insurance. The eight dimensions had binary values (1 = yes, 0 = no). Here, a composite variable of social security was constructed. That is, social security equals sick pay plus paid leave, maternity pay, medical insurance, pension insurance, working injury insurance, unemployed insurance, and maternity insurance. Also, social security was dichotomized into a binary value (1 = yes, 0 = no).

Main independent variables were age, gender, total family income, financial status, and socioeconomic status (SES). Here, age categorization was defined on the basis of the parameters of the United Nations Statistics Division (adolescent: 10-19 years; youth: 20-29 years; middle age: 30-44 years) [41] and China Health and Retirement Longitudinal Survey (old age: 45 or above; http://charls.pku.edu.cn/en/). Gender was a binary value (0 = male, 1 = female). According to standard of “Research Report of China Household Finance Survey” issued by Survey and Research Center for China Household Finance at Southwestern University of Finance and Economics (http://chfs.swufe.edu.cn/) [42], CNY < 20,000 (USD < 3006.03) was considered to be the threshold for low income group. Thus, the income group was identified using the categories of the income “CNY 0-19999 (USD 0- 3005.88) and CNY > 20,000 (USD > 3006.03)”.

Financial status denoted the question: “Compared with the other families in your rural origins, how do you rate the financial wellbeing of your family?” The option responses were categorized into three categories: not well-off, average, and well-off. The responses provided a subjective measure of family wealth. Educational level was recoded into primary education, middle education, and higher education. The respondent’s education level, number of family members, average monthly wage, household registration, and political belief of the respondent were taken as components for a three-level social class scale (upper, middle, low). This variable, SES, was treated in the analysis as a family attribute. Its calculation can be found in the Additional file 1: Table S1.

In order to explore the medical equity, the two variables were used. The dependent variable, hospital visits, denoted the question: “How many times have you seen a doctor from August 1, 2008 to July 1, 2009?” The inflated variable, abusive supervision, was referred to the question: “Did you experience forced labor, risky operation, punishing stand and kneel, body-searched & bag-searched events, managers’ hit, false imprisonment, or harmful work environment?” The option responses were dichotomized as yes (=1) and no (=0). In fact, abusive supervision also was a composite variable of forced labor, risky operation, punishing stand and kneel, body-searched & bag-searched events, managers’ hit, false imprisonment, and harmful work environment. Abusive supervision might be a good choice as inflated variable. Under abusive supervisions, the rural migrant workers were risk of the mental disorder which would worsen their health status.

In order to reflect the reimbursement rejection, the medical reimbursement were assessed with three questions: “How many work-related injuries expenses were reimbursed?”, “How many outpatient expenses were reimbursed?”, and “How many inpatient expenses were reimbursed?” Response options included: a) all were reimbursed; b) part of the medical expenses were reimbursed; and c) none of the medical expenses were reimbursed; d) did not know. The option response were dichotomized as rejected status (=1) and reimbursed status (=0).

### Main research questions

There were three questions to be explored below,

1. Were there significant associations between social class and access to social security among rural migrant workers?
2. Did social class contribute hospital visits among rural migrant workers with the dimensions of social security?
3. Were there significant associations between social security and reimbursement rejection among rural migrant workers with unfair experiences?

### Analyses

In order to answer the main research questions, the research was designed as three stages. The first stage was to explore how social inequity was associated with social security. This part aimed to show how socioeconomic factors influenced social security. Thus, the rural migrant workers were described by a set of socioeconomic variables. The characteristics of social security also were analyzed. Multivariate logistic regression models were conducted to identify factors associated with social security. Risks were expressed as adjusted odds ratios (AOR) with 95% confidence interval (95CI).

The second stage was to explore how socioeconomic factors were associated with medical inequity. If a migrant worker had one dimension of the social security, how socioeconomic factors influenced his/her hospital visits? Here, ZINB regression with Vuong test statistic was adopted to analyze the variable, hospital visits. Before using ZINB regression for hospital visits among the rural migrant workers, the mean of zero visit might point that some of rural migrant workers did not seek hospital services due to lack of social security and high medical charges. Thus, the relationship will be studied using ZINB regression among the rural migrant workers with social security.

The third stage was to reveal the relationship between the social security and reimbursement rejection. Although part of rural migrant workers had social security, they were not reimbursed medically. In order to explore the association between social security and medical reimbursement, work-related injuries expenses, outpatient expenses, and inpatient expenses operated as explained variables. In truth, the three variables were used to measure reimbursement rejection. Subsequently, multiple logistic regressions were used to explore the associations.

All the statistical analyses were performed using STATA version 14.

## Result

The present study sample was derived from the 2009 survey of 848 rural migrant workers in Pearl River Delta. Overall, 417 (49.17%) were males and 431(50.83%) were females. Mean age of the sample was 28.98 (SD = ±8.94) ranging from 13.6 to 65.5 years. Part of the sample experienced abusive supervision. Among them, 63 workers were forced to work. Twenty-seven workers worked at risk. Two workers were punished to kneel down and stand in shame. Seventeen workers were searched at the body and wallets. One worker was incarcerated. One-hundred ninety nine persons worked in the harmful environment.

Regarding work-related injuries expenses, 303 workers were totally reimbursed, 208 workers were partially reimbursed, and 164 workers were not reimbursed. Considering outpatient expenses, 46 workers were totally reimbursed, 163 workers were partially reimbursement, and 505 workers were not reimbursed. With regard to inpatient expenses, 52 workers were total reimbursed, 230 workers were partially reimbursed, and 408 workers were not reimbursed. Other workers did not know whether they were reimbursed. Thus, not all the rural migrant workers could be reimbursed medically. Average family income was CNY 39086.05 ± 58,906.76 (USD 5874.69 ± 8853.77) annually.

Table 1 presented the background characteristics. Most of them were youth and middle-aged adults and accepted middle and below education, with family income more than CNY 20,000. Over half of them were males. The numbers of SES categories were almost equal.

**Table 1:** Background characteristics, frequencies, percentage, median and inter-quartile range (IQR) for the sample

	Not well-off (%)		Average (%)		Well-off (%)	
	N (%)	Median (IQR)	N (%)	Median (IQR)	N (%)	Median (IQR)
Age group (*N* = 824)						
Adolescent	29 (3.52)	20 (10–40)k	65 (7.89)	26 (15–40)k	11 (1.33)	40 (30–50)k
Young	142 (17.23)	23.2 (18–37.5)k	222 (26.94)	35 (24–50)k	53 (6.43)	45 (30–75)k
Middle age	108 (13.11)	22 (15–30)k	115 (13.96)	30 (20–45)k	21 (2.55)	35 (20–48)k
Old age	29 (3.52)	21.36 (16–30)k	26 (3.16)	27.5 (20–40)k	3 (0.36)	200 (48–300)k
Gender (*N* = 825)						
Male	163 (19.76)	24 (15–38)k	201 (24.36)	30 (20–50)k	40 (4.85)	40 (30–75)k
Female	146 (17.70)	20 (15–31)k	227 (27.52)	30 (20–45)k	48 (5.82)	40 (24–60)k
Educational level (*N* = 825)						
Primary education	185 (22.42)	20 (14.4–30)k	247 (29.94)	27.15 (18.5–40)k	41 (4.97)	32 (23–47)k
Middle education	97 (11.76)	27 (18–40)k	134 (16.24)	37 (25–50)k	29 (3.52)	50 (30–72.5)k
Higher education	27 (3.27)	40 (20–50)k	47 (5.70)	50 (25–60)k	18 (2.18)	60 (35–150)k
Family income (*N* = 791)						
CNY 0–19,999	95 (12.01)	10 (8.5–15)k	80 (10.11)	12 (9.8–15)k	8 (1.01)	12.06 (10.5–14)k
CNY 20000-	202 (25.54)	30 (22–40)k	328 (41.47)	36 (26.25–50)k	78 (9.86)	45 (30–75)k
SES group (*N* = 797)						
Lower class	112 (14.05)	20 (13–27)k	125 (15.68)	25.75 (19–40)k	17 (2.13)	37.5 (25 –49)k
Middle class	87 (10.92)	20 (12–33.3)k	149 (18.70)	30 (20–50)k	29 (3.64)	30 (26–50)k
Upper class	104 (13.05)	30 (20–46)k	137 (17.19)	35.6 (25–60)k	37 (4.64)	55 (32.5–80)k

k = 1000. *CNY* Chinese Yuan, *SES* socioeconomic status

The results of descriptive analysis of abusive supervision, the main dimensions of social security and reimbursement rejection were presented in Table 2. As shown in Table 2, significant differences were observed in social security, the main dimensions of social security and reimbursement rejection among lower, middle, and upper class. But, no significant difference was observed in abusive supervision among the classes. More than half of the rural migrant workers were left uninsured by social security.

**Table 2:** Descriptive analysis of the main dimensions of social security, reimbursement rejection, and abusive supervision

	Lower class (%)	Middle class (%)	Upper class (%)	Chi square	*P* value
Social security (*N* = 555)				18.0575	0.000***
No	13.69	10.99	7.03		
Yes	20.00	21.26	27.03		
Sick pay (*N* = 749)				46.4290	0.000***
No	25.63	22.30	17.76		
Yes	6.54	10.68	17.09		
Paid leave (*N* = 779)				45.3200	0.000***
No	21.31	19.38	13.86		
Yes	10.65	12.97	21.82		
Maternity pay (*N* = 670)				27.9425	0.000***
No	24.48	20.30	17.46		
Yes	8.36	12.24	17.16		
Medical insurance (*N* = 771)				49.2592	0.000***
No	20.75	15.82	12.58		
Yes	10.77	16.73	23.35		
Pension insurance (*N* = 759)				77.0615	0.000***
No	25.56	20.82	15.42		
Yes	6.46	11.07	20.69		
Occupational injury insurance (*N* = 761)				25.8596	0.000***
No	18.40	13.80	13.53		
Yes	12.75	18.92	22.60		
Unemployment insurance (*N* = 731)				45.2246	0.000***
No	29.69	26.81	23.67		
Yes	2.46	6.29	11.08		
Maternity insurance (*N* = 689)				20.4153	0.000***
No	29.61	27.72	25.11		
Yes	3.19	5.66	8.71		
Reimbursement rejection					
Work-related injuries expenses (*N* = 656)				5.4141	0.067*
No	22.87	26.52	26.37		
Yes	9.60	6.71	7.93		
Outpatient expenses (*N* = 695)				10.4019	0.006***
No	6.91	9.64	12.37		
Yes	25.32	22.59	23.17		
Inpatient expenses (*N* = 673)				26.2765	0.000***
No	9.36	13.08	18.42		
Yes	23.03	19.47	16.64		
Abusive supervision (*N* = 793)				3.2743	0.195
No	21.44	22.82	26.61		
Yes	10.47	9.46	9.21		

Note: ***, ** and * indicates 1%, 5% and 10% significance level, respectively

Table 3 reported the associations between socioeconomic factors and social security. Totally, middle Age (AOR = 1.82, 95CI: 1.15-2.88) and upper class (AOR = 2.15, 95CI: 1.04–4.45) had significantly positive associations with no social security. Because significant odds were lower than 1, age categories had significantly negative associations with sick pay, paid leave, maternity pay, medical insurance, pension insurance, occupational injury insurance, unemployment insurance, and maternity insurance.

**Table 3:** Odds ratio of logistic regression model on the dimensions of social security

	Nsocialsecurity	SP	PL	MP	MI	PI	OII	UI	MAI
Age group (*N* = 824 Ref. = Adolescent()									
Young	1.46	0.71	0.93	1.00	1.10	0.74	1.26	0.39***	0.38***
Middle Age	1.82**	0.57***	0.70*	0.57***	1.01	0.62**	1.24	0.36***	0.27***
Old Age	0.75	0.33***	0.31***	0.11***	0.25***	0.37***	0.59*	0.08***	0.09***
Gender (*N* = 825 Ref. = Male)									
Female	0.98	0.68**	0.91	1.43**	0.85	0.88	0.70**	0.62***	0.94
Family income (*N* = 791 Ref.=)									
CNY 0-19,999									
CNY 20000-	1.26	0.84	1.05	0.81	0.90	0.73*	1.23	0.98	0.77
Educational level (*N* = 825 Ref.=)									
Primary education									
Middle education	0.96	0.95	1.17	1.16	1.33	1.85**	1.29	2.39***	1.65
Higher education	1.54	1.88*	1.83*	3.32***	2.34**	4.30***	2.54***	9.70***	5.64***
Financial status									
Not well-off									
Average	0.89	0.80	0.82	0.60***	0.79	0.76	0.71**	0.57***	0.71
Well-off	0.81	0.93	0.83	0.60*	0.69	0.72	0.50**	0.58	0.69
SES group (*N* = 797 Ref. = Lower class)									
Middle class	1.29	1.09	0.89	0.99	1.32	0.97	1.42*	0.76	0.70
Upper class	2.15**	1.94**	1.53	1.10	1.74**	1.40	1.19	0.54	0.60

Note: ***, ** and * indicates 1%, 5% and 10% significance level, respectively. *SP* sick pay, *PL* paid leave, *MP* maternity pay, *MI* medical insurance, *PI* pension insurance, *OII* occupational injury insurance, *UI* unemployment insurance, *MAI* maternity insurance, *CNY* Chinese Yuan, *SES* socioeconomic status

Female had significantly positive associations with maternity pay (AOR = 1.43, 95CI: 1.03–1.98), while it had significantly negative associations with sick pay (AOR = 0.68, 95CI: 0.50–0.92), occupational injury insurance (AOR = 0.70, 95CI: 0.52–0.94), and unemployment insurance (AOR = 0.62, 95CI: 0.44–0.88).

With respect to education, middle education had significantly positive associations with pension insurance (AOR = 1.85, 95CI: 1.15–2.97) and unemployment insurance (AOR = 2.39, 95CI: 1.28–4.45). Higher education had significantly positive associations with sick pay (AOR =1.88, 95CI: 0.94–3.79), paid leave (AOR = 1.83, 95CI: 0.93–3.61), maternity pay (AOR = 3.32, 95CI: 1.53–7.22), medical insurance (AOR =2.34, 95CI: 1.17–4.68), pension insurance (AOR = 4.30, 95CI: 2.11–8.78), occupational injury insurance (AOR = 2.54, 95CI: 1.26–5.10), unemployment insurance (AOR = 9.70, 95CI: 4.08–23.04), and maternity insurance (AOR = 5.64, 95CI: 2.32–13.72).

Regarding financial status, average status had significantly negative associations with maternity pay (AOR = 0.60, 95CI: 0.42–0.84), occupational injury insurance (AOR = 0.71, 95CI: 0.51–0.98), and unemployment insurance (AOR = 0.57, 95CI: 0.39–0.83). Well-off status had significantly negative associations with maternity pay (AOR = 0.60, 95CI: 0.33–1.10) and occupational injury insurance (AOR = 0.50, 95CI: 0.29–0.87).

Regarding SES group, middle class had significantly positive associations with occupational injury insurance (AOR = 1.42, 95CI: 0.97–2.07). Upper class had significantly positive associations with sick pay (AOR = 1.94, 95CI: 1.10–3.42) and medical insurance (AOR = 1.74, 95CI: 1.02–2.97).

Thus, question 1 was answered.

**Fig. 2:**
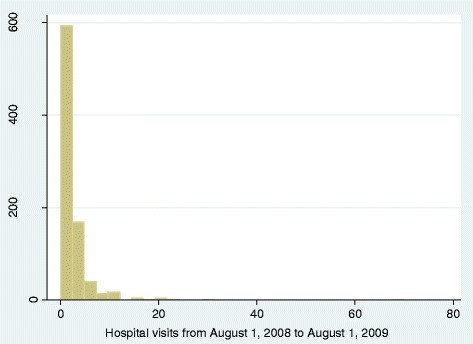
The distribution of hospital visits

See Fig. 2. The distribution of hospital visits satisfied the requirements of ZINB regression that hospital visits had 118 zeros among available 841 observations.

The associations between socioeconomic factors and hospitals visits among rural migrant workers with social security can be seen in Table 4. When the rural migrant workers were covered by sick pay, paid leave, maternity pay, occupational injury insurance, and maternity insurance, middle age significantly made positive contribution to hospitals visits. When the rural migrant workers were covered by paid leave, medical insurance, and occupational injury insurance, respectively, old age significantly made positive contribution to hospitals visits. When rural migrant workers were covered by paid leave, maternity pay, medical insurance, pension insurance, and occupational injury insurance, female significantly made positive contribution to hospitals visits. When the rural migrant workers were covered by sick pay, paid leave, maternity pay, medical insurance, pension insurance, occupational injury insurance, and unemployment insurance, average financial status significantly made negative contribution to hospitals visits. When the rural migrant workers were covered by maternity pay, medical insurance, pension insurance, and unemployment insurance, well-off financial status significantly made negative contribution to hospitals visits. When rural migrant workers were covered by maternity pay, middle class significantly made negative contribution to hospitals visits. Upper class significantly made positive contribution to hospitals visits when rural migrant workers were covered by sick pay. Here, abusive supervision did not significantly contribute to hospitals visits. Additionally, all the coefficients of abusive supervision were negative. It could speculate that abusive supervision undermined the diagnostic motivations of workers. Thus, question 2 was answered.

**Table 4:** Zero-inflated negative binomial regression for hospital visits among rural migrant workers with social security

	SP = 1	PL = 1	MP =1	MI = 1	PI = 1	OII = 1	UI = 1	MAI = 1
Age group (*N* = 824 Ref. = Adolescent)								
Young	0.35 (0.25)	0.25 (0.21)	0.51 (0.31)	−0.06 (0.22)	−0.32 (0.28)	0.13 (0.22)	0.44 (0.49)	0.21 (0.36)
Middle Age	0.62 ** (0.25)	0.50** (0.22)	0.75** (0.32)	0.28 (0.22)	−0.03 (0.28)	0.40* (0.22)	0.59 (0.49)	0.62* (0.36)
Old Age	0.40 (0.36)	0.80** (0.32)	0.66 (0.58)	1.09*** (0.33)	0.33 (0.36)	0.78*** (0.29)	0.24 (0.72)	0.50 (0.55)
Gender (*N* = 825 Ref. = Male)								
Female	0.17 (0.12)	0.29** (0.11)	0.29** (0.13)	0.29 *** (0.11)	0.43*** (0.13)	0.29*** (0.10)	0.21 (0.16)	0.07 (0.16)
Family income (*N* = 791 Ref. = CNY 0-19,999)								
CNY 20000-	−0.12 (0.16)	−0.00 (0.14)	0.16 (0.15)	0.04 (0.14)	0.24 (0.16)	0.00 (0.13)	0.10 (0.25)	−0.23 (0.21)
Educational level (*N* = 825 Ref. = Primary education)								
Middle education	−0.09 (0.18)	0.20 (0.17)	−0.13 (0.19)	0.06 (0.16)	0.12 (0.18)	0.04 (0.15)	−0.19 (0.24)	0.09 (0.23)
Higher education	−0.19 (0.23)	0.03 (0.23)	−0.13 (0.24)	−0.07 (0.22)	−0.16 (0.24)	−0.13 (0.21)	−0.22 (0.32)	0.40 (0.28)
Financial status (*N* = Ref. = Not well-off)								
Average	−0.24* (0.13)	−0.23* (0.12)	−0.37*** (0.13)	−0.31*** (0.11)	−0.33** (0.13)	−0.21* (0.11)	−0.27* (0.16)	−0.16 (0.16)
Well-off	0.12 (0.18)	−0.14 (0.19)	−0.62*** (0.22)	−0.45** (0.19)	−0.74*** (0.22)	−0.21 (0.18)	−0.57** (0.28)	−0.42 (0.27)
SES group (*N* = 797 Ref. = Lower class)								
Middle class	0.14 (0.19)	0.02 (0.16)	−0.35** (0.18)	0.07 (0.15)	−0.04 (0.20)	0.04 (0.14)	−0.19 (0.25)	0.17 (0.24)
Upper class	0.49** (0.23)	0.00 (0.21)	0.00 (0.23)	0.12 (0.20)	−0.02 (0.24)	0.14 (0.19)	−0.09 (0.32)	0.25 (0.29)
Intercept	0.26 (0.30)	0.43* (0.26)	0.32 (0.35)	0.65** (0.25)	0.83** (0.33)	0.50** (0.25)	0.55 (0.54)	0.39 (0.43)
Inflate Abusive supervision	−0.18 (5289.03)	−0.22 (20,938.75)	−0.91 (78,237.29)	−0.32 (6134.49)	−0.36 (7380.20)	−0.33 (16,561.18)	−0.44 (9292.63)	−0.27 (14,218.81)
Intercept	−20.79 (3577.86)	−23.36 (12,385.97)	−25.07 (48,867.37)	−21.30 (5119.98)	−21.49 (6159.40)	−23.53 (13,618.64)	−21.09 (6398.96)	−21.66 (8274.09)
/lnalpha	−1.13 *** (0.20)	−0.65*** (0.13)	−1.03*** (0.19)	−0.71*** (0.14)	−0.73*** (0.15)	−0.74*** (0.13)	−1.29*** (0.30)	−2.70 *** (0.91)
Alpha	0.32 (0.06)	0.52 (0.07)	0.36 (0.07)	0.49 (0.07)	0.48 (0.07)	0.48 (0.06)	0.27 (0.08)	0.07 (0.06)

Note: ***, ** and * indicates 1%, 5% and 10% significance level, respectively. *SP* sick pay, *PL* paid leave, *MP* maternity pay, *MI* medical insurance, *PI* pension insurance, *OII* occupational injury insurance, *UI* unemployment insurance, *MAI* maternity insurance, *CNY* Chinese Yuan, *SES* socioeconomic status

Table 5 reported the associations between reimbursement rejection and access to social security. In the case of work-related injuries expenses in the AS group, pension insurance and occupational injury insurance had significantly negative associations with reimbursement rejection, while unemployment insurance had significantly positive association with reimbursement rejection. At the same time in the Non-AS group, maternity pay had significantly positive association with reimbursement rejection, while sick pay and medical insurance had significantly negative associations with reimbursement rejection. Also, implementation of occupational injury insurance had significantly negative association with reimbursement rejection in both AS and Non-AS group.

**Table 5:** Logistic regression models of the social security on reimbursement rejection

	Work-related injuries expenses				Outpatient expenses				Inpatient expenses			
	AS		Non-AS		AS		Non-AS		AS		Non-AS	
	AOR	95CI	AOR	95CI	AOR	95CI	AOR	95CI	AOR	95CI	AOR	95CI
Sick pay (Ref. = No)												
Yes	1.21	0.11–13.62	0.28***	0.11–0.72	0.63	0.14–2.79	0.83	0.47–1.47	0.31	0.07–1.36	0.81	0.45–1.47
Paid leave(Ref. = No)												
Yes	0.29	0.04–1.91	0.94	0.48–1.84	1.36	0.43–4.27	3.01***	1.78–5.07	0.77	0.26–2.25	2.11***	1.26–3.53
Maternity pay (Ref. = No)												
Yes	0.33	0.05 –2.02	2.22*	0.92–5.38	0.32	0.06–1.63	0.97	0.50–1.88	0.21	0.03–1.42	0.89	0.46–1.71
Medical insurance(Ref. = No)												
Yes	0.63	0.07–5.61	0.28**	0.10–0.81	0.54	0.15–1.97	0.75	0.39–1.44	0.25*	0.06–1.08	0.40**	0.20–0.82
Pension insurance (Ref. = No)												
Yes	0.05**	0.00–0.83	2.21	0.64–7.62	4.53*	0.96–21.33	0.65	0.28–1.49	1.91	0.32–11.29	0.65	0.28–1.49
Occupational injury insurance (Ref. = No)												
Yes	0.05**	0.00–0.83	0.10***	0.04–0.26	3.68**	1.23–11.04	1.47	0.85–2.54	9.91***	2.87–34.23	1.53	0.86–2.73
Unemployment insurance (Ref. = No)												
Yes	12.92*	0.83–201.38	0.75	0.18–3.02	0.17*	0.02–1.22	0.44*	0.19–1.00	1.53	0.18–13.24	0.58	0.25–1.34
Maternity insurance (Ref. = No)												
Yes	9.50	0.48–187.03	0.98	0.25–3.82	1.10	0.18–6.76	1.55	0.70–3.44	0.17*	0.02–1.24	1.27	0.57–2.82
Log pseudolikelihood	−37.48		−146.71		−73.557		−238.37		−62.546		−230.30	
Obs	107		350		117		364		112		353	

Note: ***, ** and * indicates 1%, 5% and 10% significance level, respectively. *AS* abusive supervision, *AOR* adjusted odds ratio, *CI* confidence intervals

In the case of outpatient expenses, implementation of pension insurance and occupational injury insurance had significantly positive associations with reimbursement rejection in the AS group, while paid leave had significantly positive association with reimbursement rejection in the Non-AS group. Also, implementation of unemployment insurance had significantly negative associations with reimbursement rejection in both AS and Non-AS group.

In the case of inpatient expenses in the AS group, occupational injury insurance had significantly positive associations with reimbursement rejection, while maternity pay had significantly negative association with reimbursement rejection. At the same time in the Non-AS group, paid leave had significantly positive association with reimbursement rejection. Also, implementation of medical insurance had significantly negative association with reimbursement rejection in both AS and Non-AS group. Thus, question 3 was answered.

## Discussion

Based on the sample of rural migrant workers, this study made an effort to discover the healthcare inequity on the basis of social inequity. As the lower class in urban China, about half of the sample was left uninsured and faced high financial risk from inadequate health care, which continued to pose a huge challenge to health equity and social justice. Also, the findings here revealed the effects of demographic factors and socioeconomic status on social security among rural migrant workers in Pearl River Delta in China. Thus, the marginalized status of social class could worsen their access to social security. Seemingly, it was difficult for the workers with lower and middle class to benefit from social security. This can be explained that legal healthcare expenditure released by employers were put aside due to unaffordable costs of fighting for legitimate interests among rural migrant workers. Also, rural migrant workers with high class were significantly more likely than persons with middle class to go to hospital for diseases. It could be speculated that there was medical affordability gaps among the rural migrant workers.

In agreement with the prior studies, this study highlighted the role of socioeconomic factors in the social security. Especially, insurance participation rate differed from income levels in rural China [43, 44]. Income was one the most important factors in hospital care [45, 46] and contributed to inequity in general health care utilization [47, 48]. Thus, socioeconomic factors were crucial to symbolize social, healthcare, and reimbursement equity to medical care for the rural migrant workers.

Also, this study deepened the knowledge of the relationship between social class inequalities and inequalities to health services [49, 50]. This study was in line with the situation in Shenzhen city that the health insurance system was inequitably distributed among the rural migrant workers. Younger and less educated women who were paid less were more likely to be uninsured and therefore to pay out of pocket for their care [51]. This suggested that part of the firms did not offer social security to the rural migrant workers. Thus, there existed inequitable access to social security. Although inequitable distribution of government health care subsidies was reduced in rural outpatient services [52], wealthier people benefited more than poorer people [53]. Thus, the accepted explanation may be the gaps of social positions.

Importantly, not all the dimensions of social security had significant associations with reimbursement rejection. This suggested that the employers might offer superficial social security to the rural migrant workers in order to cope with governmental investigation. In China’ settings, social security could be quite a significant additional cost for employers although it was mandatory. Among them, pension, medical insurance, unemployment insurance, and occupational injury insurance operated by receiving contributions on a monthly basis from both the employee and the employer. But, contributions to maternity insurance were made by employers only. With respect to occupational injury insurance, the employer would still need to pay the salary to the employee during the recuperation period. Regarding sick pay, paid leave, and maternity pay, the employers should pay the salary during the period of leave from the company. Also, the benefits of migrants were neglected in the Chinese society. Urban locals possessed the urban medical welfare resources which the migrant workers could not get access to. Due to migration, it was also difficult for the employed workers to transfer medical welfare resources from rural place to urban place.

Here, hospital visits was important indicator of medical equity for the migrant workers with diseases. The migrant workers were often at great risk of illness and less likely to have medical insurance. In truth, an urban local worker with diseases would have great difficulties in getting access to essential health care due to high price of pharmaceutical and medical services. Thus, the migrant workers without coverage of health-related insurance had to pay for medical fees out-of-pocket. Facing high price of medical expenses, it often happened that some of the rural migrant workers in urban China delayed the preventive services for minor illness and withstood serious illness from curative services.

There seemed to be medical discrimination against the rural migrant workers. With respect to occupational injury insurance, it was common sense that when the workers experienced abusive supervision, their medical expenses possibly could be reimbursed. But, the statistical results reported the workers with abusive experiences were rejected by medical reimbursement of outpatient expenses and inpatient expenses. A conjectural explanation was that all the expenses covered by occupational injury insurance could not be reimbursed completely. In fact, occupational injury insurance did not cover costs in all major diseases. Similarly, medical insurance had not relieved the all financial burden of disease-related medical costs. Also, reimbursement method of health insurance would influence the interviewees’ responses. Often, immediate reimbursement rather than later reimbursement significantly increased the likelihood of seeking medical treatment among the migrant workers. Another explanation was the employers secretly rejected to reimburse high price of prescription medicines in the end. Attentively, the workers with maternity pay did experience reimbursement rejection of work-related injuries expenses. This could be guessed there was sex discrimination against the rural migrant workers in the case of abusive supervision.

The current study reported that there existed inherent and external source of inequity to the rural migrant workers. The first source was from their social status, while the second source resulted from external disregard. Sadly, socioeconomic factors influenced hospital visits among the rural migrant workers having social security. Thus, it could be speculated that there existed medical discrimination in the Chinese hospitals. Most of the rural migrant workers were the socioeconomically disadvantaged. They tended to mistrust community health service centers [54] due to marginalized status. In addition, health workforce inequity in quality and geographic distribution [55] and differences in the availability of medical care to urban and rural communities [56] could not satisfy the need of the rural migrant workers. So, compared with permanent residents of cities, rural migrant workers used health care to a lower extent [57]. More importantly, the employing firms contributed to healthcare inequity, medical inequity, and reimbursement inequity among the rural migrant workers. In fact, some companies would not pay the cost if the injured workers’ family did not appeal for and complain with the working injuries. Even more worse, normal wage of the migrant workers in urban China possibly often could be unpaid. Chinese Premier Wen Jiabao helped a rural migrant worker get back his unpaid wage. In the eyes of the Chinese workers, a common person would obtain fair treatment with a tower of strength. This may be true in most cases.

Considering policy design and implementation, this study suggested that China cannot achieve the medical equity until all the rural migrant workers could be insured and reimbursed completely. Obviously, the current gaps of the health insurance system in China reflected the principle of justice insufficiently. Clinically for this situation, the argument of Saloner and Daniels (2011), an affordable health insurance kept people functioning normally and it protected their financial security [58], had been demonstrated by physician decision-making [59, 60] and became true in Chinese settings. Based on the current social security system in China, equity in health care demanded promoting accessibility and availability to affordable health insurance, especially for poverty-stricken rural migrant workers. At the angle of health care financing and cost reform, China should expand the coverage of social security and reduce the unethical treatment of migrant class. The central government in China designed the current social security system, while local governments implemented the system. The sustainability, reform, and fund of the system also need governmental resources. China government could design social security at regular time for temporary rural migrant workers just because of weak portability of social health insurance [61]. With regard to temporary migration, weekly and monthly offer of social security should be planned, especially for the pregnant women and workers with major diseases. In the case of the family income, urban government should finance the unaffordably insured because medical fees were often higher for the poor without insurance. Also, urban hospitals could reduce and exempt medical fees for the poor and vulnerable migrants. More importantly, the firms and enterprises without social responsibilities to rural migrant workers should be punished.

Two future directions can be explored. Before 2000, the class measure was subject to ideological character [62] and work conditions [63]. But till now, no previous research has reported how to measure social class scientifically. This may be a new research direction. Another future direction was China’s health care reform. Although the reform aimed to counteract inequity of health care utilization, the reform failed to reach the beforehand expectation [64–66]. Also, the reform failure had strengthened inequitable health care utilization and health outcomes [67–69]. The China’s medical reform need be deepened with respect to the rural migrant workers.

This study also had three strengths. First, this study confirmed the neglected medical benefits of rural migrant workers in urban China with a series of statistical analysis. Second, the present study had the large sample size (*n* = 848), enabling to conduct binary logistic regressions to reveal multiple associations. A third strength was clear and efficient research design. Here, a series of regressions were adopted to find the associations step by step in the cross-sectional data.

The current study had three limitations. The first was the data used was from a self-reported questionnaire in the specific region and year. This might affect the results’ external validity. The second was scientific concept of social class was not defined. Here, the subjective and speculated considerations were adopted. Thus, the final limitation was the causal relationships between social class and social security were not discovered directly.

## Conclusion

In conclusion, the present study demonstrated that socioeconomic factors were the main determinants of inequity to social security, medical inequity, healthcare, and reimbursement among the rural migrant workers. Migrant workers were in a vulnerable state when they attempted to access to primary care services. It also confirmed that persons with low social class had difficulties in accessing to social security. Even if the rural migrant workers were offered to social security, they received limited benefits from the social security. Therefore, the findings here supported to use compulsory policies to expand the coverage of social security. In practice, China government should make a substantial effort to strengthen policy implementation in improving the income distribution and reducing the inequality to social security,health care, and medical reimbursement for the vulnerable population.

## Additional file

Additional file 1: Table S1. Proposed updation of Kuppuswamy classification of socioeconomic status. (DOCX 17 kb)

## Abbreviations

AOR: Adjusted odds ratio
AS: Abusive supervision
CI: Confidence intervals
CNY: Chinese Yuan
IQR: Interquartile range
MAI: Maternity insurance
MI: Medical insurance
MP: Maternity pay
OII: Occupational injury insurance
PI: Pension insurance
PL: Paid leave
SES: Socioeconomic status
SMWPRD: 2009 Survey of Migrant Workers in Pearl River Delta
SP: Sick pay
UI: Unemployment insurance
USD: United States dollar
ZINB regression: Zero-inflated negative binomial regression
