# Supplementary material for: Should the poor have no medicines to cure? A study on the association between social class and social security among the rural migrant workers in urban China
Source: Int J Equity Health. 2017 Nov 7;16:193. doi: 10.1186/s12939-017-0692-x (PMC5678794; doi:10.1186/s12939-017-0692-x)
Supplement: Additional file 1: Table S1. — Proposed updation of Kuppuswamy classification of socioeconomic status. (DOCX 17 kb) [file 12939_2017_692_MOESM1_ESM.docx]

Additional File 1: Table S1. Proposed updation of Kuppuswamy classification of socioeconomic status

| **Education level** | **Score** |
| --- | --- |
| No formal education | 1 |
| Primary school | 2 |
| Junior middle school | 3 |
| Senior middle school | 4 |
| Technical secondary school | 5 |
| Vocational (technical) school | 6 |
| Junior college | 7 |
| **Number of family members** |  |
| 2-4 | 3 |
| 5-9 | 2 |
| 10- | 1 |
| **Average monthly wage** |  |
| 500-860 | 1 |
| 861-1550 | 2 |
| 1551- | 3 |
| **Household registration (Hukou)** |  |
| Nonagricultural Hukou | 7 |
| Agricultural Hukou | 4 |
| Without Hukou | 1 |
| **Political belief of the respondent** |  |
| Democratic party | 7 |
| Communist Party of China | 5 |
| Communist youth league | 3 |
| General public | 1 |
| **Class Category** |  |
| Upper class | Above 16 |
| Middle class | 13-15 |
| Lower class | Below 12 |

Note: CNY 860 and 1550 (USD 129.23 and 232.98) were two cutoffs from upper limits of minimum wage standard in 2008 and 2016 in Guangdong Province in China categarised average monthly wage into three groups (lower,middle,and high).
